# Hypoxia and oxidative stress induce sterile placental inflammation in vitro

**DOI:** 10.1038/s41598-021-86268-1

**Published:** 2021-03-31

**Authors:** Bernadette C. Baker, Alexander E. P. Heazell, Colin Sibley, Rachael Wright, Helen Bischof, Frances Beards, Tatiana Guevara, Sylvie Girard, Rebecca L. Jones

**Affiliations:** 1 Maternal and Fetal Health Research Centre, Division of Developmental Biology and Medicine, School of Medical Sciences, Faculty of Biology, Medicine and Health, University of Manchester, Oxford Road, Manchester, M13 9WL UK; 2 St Mary’s Hospital, Central Manchester NHS Foundation Trust, Manchester Academic Health Science Centre, Manchester, M13 9WL UK; 3 Fetomaternal and Neonatal Pathologies Research Axis, Sainte-Justine Hospital Research Center, Montreal, Canada; 4 Department of Obstetrics and Gynecology, Universite de Montreal, Montreal, QC Canada

**Keywords:** Developmental biology, Immunology, Diseases, Medical research, Pathogenesis

## Abstract

Fetal growth restriction (FGR) and stillbirth are associated with placental dysfunction and inflammation and hypoxia, oxidative and nitrative stress are implicated in placental damage. Damage-associated molecular patterns (DAMPs) are elevated in pregnancies at increased risk of FGR and stillbirth and are associated with increase in pro-inflammatory placental cytokines. We hypothesised that placental insults lead to release of DAMPs, promoting placental inflammation. Placental tissue from uncomplicated pregnancies was exposed in vitro to hypoxia, oxidative or nitrative stress. Tissue production and release of DAMPs and cytokines was determined. Oxidative stress and hypoxia caused differential release of DAMPs including uric acid, HMGB1, S100A8, cell-free fetal DNA, S100A12 and HSP70. After oxidative stress pro-inflammatory cytokines (IL-1α, IL-1β, IL-6, IL-8, TNFα, CCL2) were increased both within explants and in conditioned culture medium. Hypoxia increased tissue IL-1α/β, IL-6, IL-8 and TNFα levels, and release of IL-1α, IL-6 and IL-8, whereas CCL2 and IL-10 were reduced. IL1 receptor antagonist (IL1Ra) treatment prevented hypoxia- and oxidative stress-induced IL-6 and IL-8 release. These findings provide evidence that relevant stressors induce a sterile inflammatory profile in placental tissue which can be partially blocked by IL1Ra suggesting this agent has translational potential to prevent placental inflammation evident in FGR and stillbirth.

## Introduction

Fetal growth restriction (FGR) describes the failure of the fetus to reach its growth potential and is a major risk factor for perinatal morbidity and mortality^1^. Whilst improvements in neonatal care have improved outcomes for growth restricted infants and those born prematurely due to iatrogenic delivery, stillbirth rates have improved only modestly over the past 20 years^2^. This is largely due to a lack of understanding of the underlying pathogenic mechanisms, hampering clinical advances. Detection and management of pregnancies at high risk of FGR and stillbirth remains a major challenge in maternity care^3^ and to date there are no effective drug therapies, with early delivery the only option to prevent stillbirth.

Both FGR and stillbirth are strongly associated with placental dysfunction, a term which encompasses a range of abnormalities in the development, growth and function of the placenta, leading to an inability to sustain fetal growth and in some cases viability^4,5^. The causes of placental dysfunction are unknown, but there is evidence for placental hypoxia and oxidative/nitrative stress in pregnancies complicated by FGR, attributed to abnormalities in placentation and subsequent aberrant uteroplacental perfusion^6–9^. However, the mechanistic pathways linking hypoxia, oxidative stress and placental dysfunction have not been fully elucidated.

We recently found evidence of placental inflammation in high-risk pregnancies and pregnancies ending in stillbirth^10–13^. Women reporting a reduction in fetal movements (RFM) in the third trimester of pregnancy have a threefold increased risk of delivering a growth restricted or stillborn infant^14^. These pregnancies are characterised by placental dysfunction; the presence of histopathological findings of maternal vascular malperfusion and inflammation is consistent with the theory that fetal activity is reduced to conserve energy due to insufficient oxygen and nutrient supply^15,16^. Placentas from RFM pregnancies have elevated pro-inflammatory and reduced anti-inflammatory cytokines, indicating a pro-inflammatory bias which is mirrored in the maternal circulation^10^. No clinical signs of infection were apparent in these pregnancies, but elevated levels of DAMPs (danger associated molecular patterns or alarmins) were detected in maternal blood. DAMPs are endogenous intracellular factors—lipids, nucleic acids, metabolites or proteins—which when released or secreted following cell damage, death or stress, can trigger non-pathogenic inflammation^17–19^. This occurs through activation of similar inflammatory pathways as pathogens and can exacerbate local cell and tissue dysfunction due to tissue damage or subclinical infection.

In RFM pregnancies, we found three DAMPs elevated in maternal circulation, uric acid, cell-free fetal DNA (cffDNA) and high-mobility group box 1 (HMGB1)^10^. Two further cohort studies have shown a consistent pattern of DAMPs and pro-inflammatory cytokines altered in the maternal and placental compartments. A study comparing plasma from 200 women in Canada with normal or pathological pregnancies identified increased circulating HMGB1 and IL1α in the second trimester of women who went on to have an FGR infant^13^ and in the UK elevated circulating S100A8 was found in third trimester maternal serum along with elevated uric acid and IL-8 content in placentas from fetuses with a significantly reduced growth rate^12^.

The origin and cause of elevated circulating DAMPs is unknown, but placental cells express and can release several DAMPs in vitro^20–23^. Previous studies have examined individual or limited numbers of DAMPs or cytokines in short term placental explant culture or cell lines under hypoxia or oxidative stress^24–28^ demonstrating the principle that hypoxia and oxidative stress can increase the levels of DAMPs and pro-inflammatory cytokines (Supplementary Table 1). However, to date a systematic analysis of multiple DAMPS and cytokines produced by the placenta under stress conditions has not been performed. Term human placental explants derived from normal pregnancies are a well-established ex vivo culture model which retain tissue architecture, cell interactions and functionality of intact placenta^29^. This model has been used to explore transport, metabolism, endocrine and immune functions^30–32^ and allows in vitro conditions to be easily manipulated to determine altered responses which are impossible to achieve in vivo.

We hypothesized that placental damage results in the release of DAMPs, which stimulates or exacerbates placental inflammation. The aims of this study were to determine whether placental insults that mimic those present in pregnancy complications (hypoxia, oxidative stress, nitrative stress) result in the release of DAMPs from the placenta and subsequent placental inflammation. This would aid in understanding the contribution of the placenta as a potential source of these DAMPs/cytokines and how this could relate to observed changes in pathological pregnancies.

An in vitro model of term placental explants was employed to assess the effects of placental damage on DAMP and cytokine release and interrogate the underlying mechanistic pathways whilst exploring whether anti-inflammatory therapies have translational potential as a treatment to reduce placental inflammation.

## Results

### Placental insults induce DAMP release from placental explants

**Figure 1:**
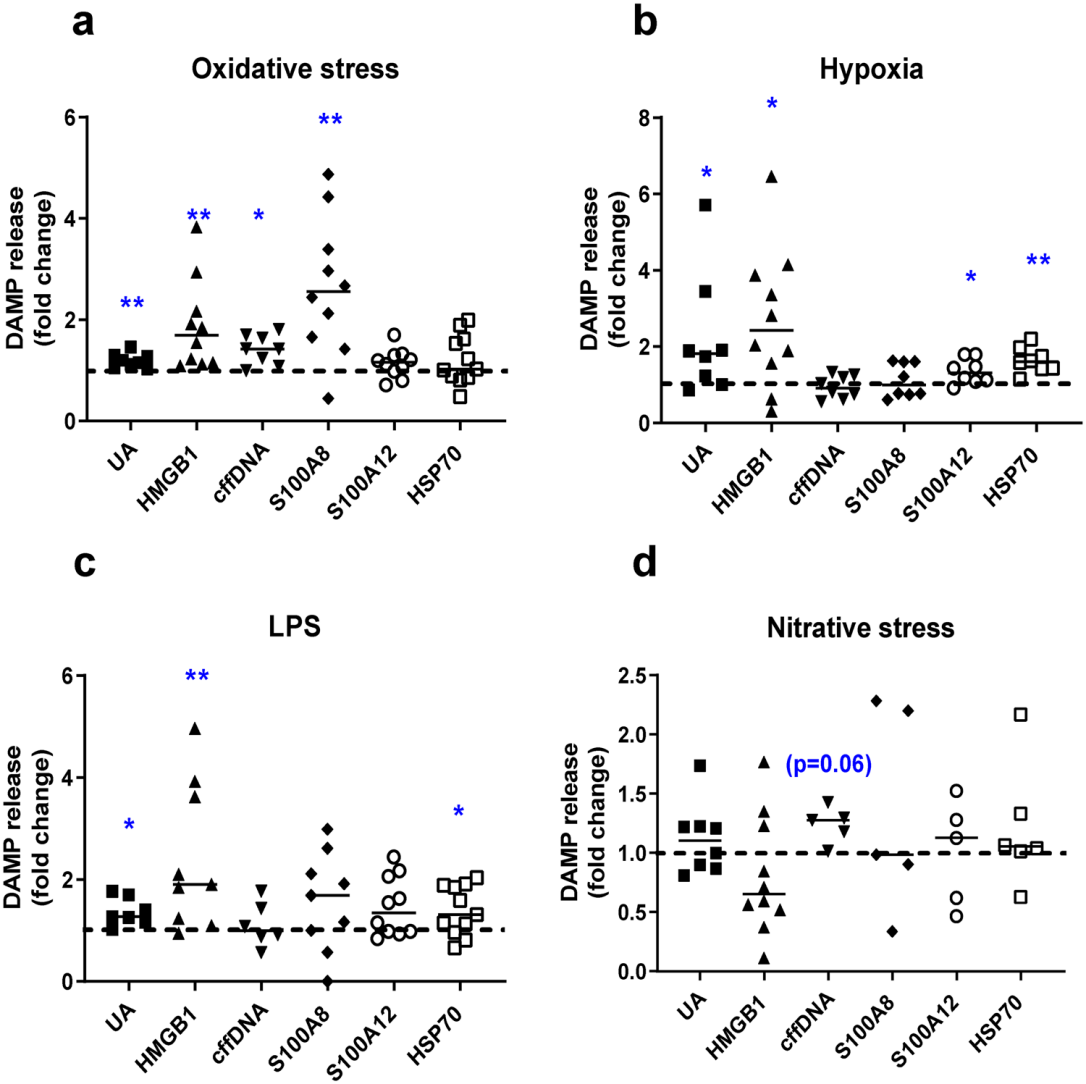
Effect of placental insults on release of DAMPs by term placental explants. Explants were exposed for 72h to (**a**) oxidative stress (1 mM H_2_O_2_), (**b**) hypoxia (1%O_2_), (**c**) low dose LPS (1 ng/ml to mimic subclinical infection), (**d**) nitrative stress (1 mM SIN-1). Concentrations of uric acid (UA), high mobility group box 1 (HMGB1), cell free fetal DNA (cffDNA), S100 calcium binding proteins A8 (S100A8) and A12 (S100A12), heat shock protein 70 (HSP70) were measured in culture medium and corrected for explant protein content. Data are fold change from control conditions (n = 5–11, line represents median); dashed line represents control values. *p < 0.05, **p < 0.01 Wilcoxon signed rank.

Established in vitro models of placental hypoxia, oxidative stress, nitrative stress and low level infection^32–35^ were utilised to determine the effect of pathologically relevant placental insults on DAMP release (Fig. 1 and Supplementary Table 2). Markers of tissue viability including explant cell proliferation, apoptosis, β-hCG hormone release and necrosis were assessed. Changes in these markers agreed with previously published observations in these models and confirmed that explant integrity was maintained (Supplementary Fig. 2)^32,34,36,37^. Evidence of nitrative stress by significantly increased levels of nitrotyrosine in placental explants exposed to 1 mM SIN1was confirmed (Supplementary Fig. 3).

Uric acid and HMGB1 concentrations were elevated in culture medium following hypoxia, oxidative stress and low level LPS treatment (*p* < 0.05–0.01). S100A8 was elevated following exposure to oxidative stress (*p* < 0.01), whilst both HSP70 and S100A12 were elevated in response to hypoxia only (*p* < 0.05). cffDNA levels were increased following exposure to oxidative stress (*p* < 0.05) with a trend towards elevated levels following exposure to nitrative stress (*p* = 0.06).

**Figure 2:**
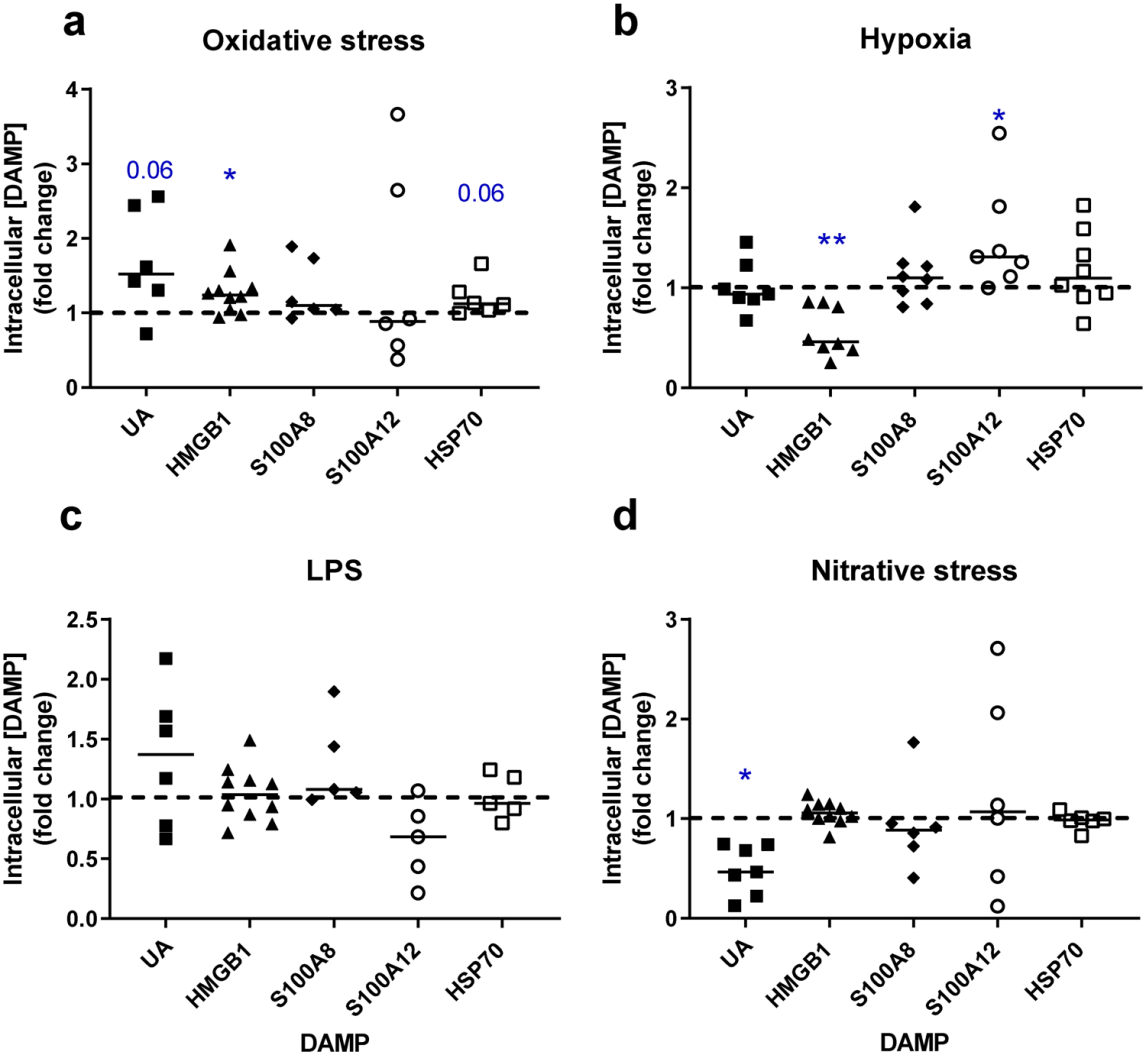
Effect of placental insults on intracellular concentrations of DAMPs in term placental explants. Explants were exposed for 72h to (**a**) oxidative stress (1 mM H_2_O_2_), (**b**) hypoxia (1%O_2_), (**c**) low dose LPS (1 ng/ml to mimic subclinical infection), (**d**) nitrative stress (1 mM SIN-1). Concentrations of uric acid (UA), high mobility group box 1 (HMGB1), S100 calcium binding proteins A8 (S100A8) and A12 (S100A12), heat shock protein 70 (HSP70) were measured in placental lysates and corrected for explant protein content. Data are fold change from control conditions (n = 5–10, line represents median); dashed line represents control values. *p < 0.05, **p < 0.01 Wilcoxon signed rank test.

DAMP concentrations were assessed in placental lysates to determine whether the increased levels of DAMPs in culture medium were due to elevated production or release of intracellular contents (Fig. 2 and Supplementary Table 1). Differential effects on HMGB1 were observed, with lower intracellular levels following exposure to hypoxia (*p* < 0.01) but elevated in response to oxidative stress (*p* < 0.05). A similar trend towards increases in intracellular UA and HSP70 concentration was observed in response to oxidative stress (*p* = 0.06). Uric acid concentrations were lower in response to nitrative stress (*p* < 0.05). Intracellular levels of S100A8 and A12 were unaffected by oxidative or nitrative stress whereas hypoxia increased S100A12 only. Treatment with LPS had no effect on intracellular DAMP concentrations.

### Placental insults induce cytokine production and release from placental explants

**Figure 3:**
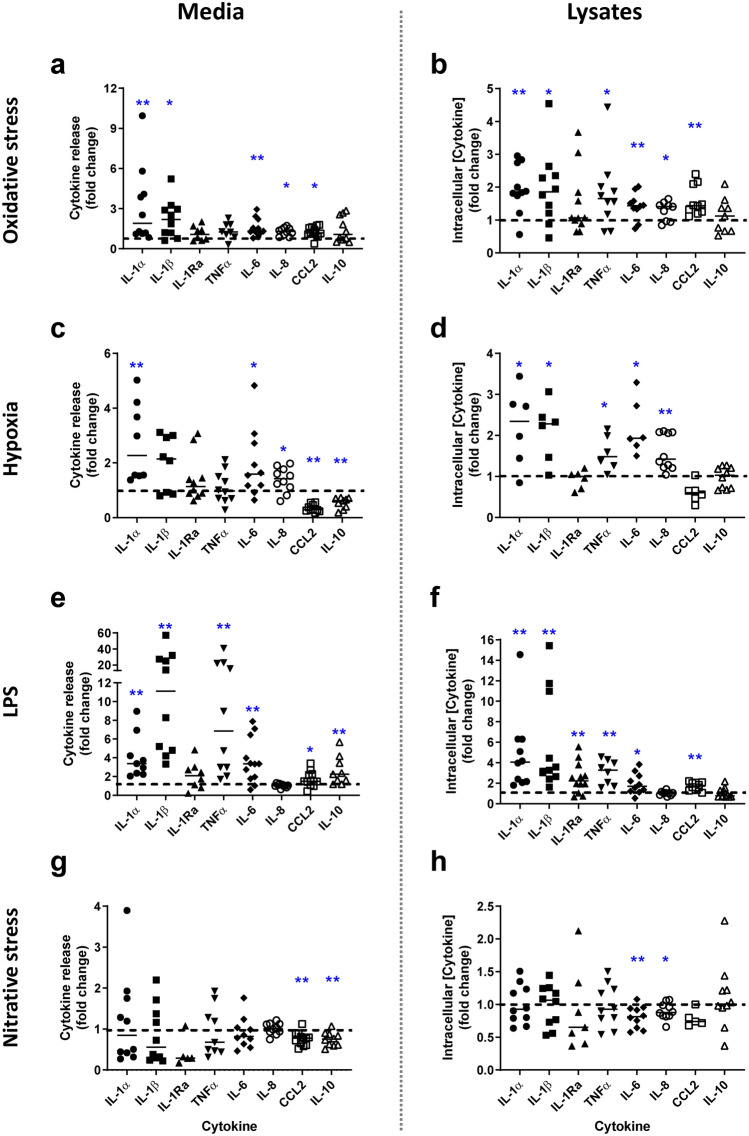
Effect of insults on term placental explant cytokine release and intracellular concentrations. Explants were exposed to (**a** and **b**) oxidative stress (1 mM H_2_O_2_), (**c** and **d**) hypoxia (1%O_2_), (**e** and **f**) low dose LPS (1 ng/ml to mimic subclinical infection), (**g** and **h**) nitrative stress (1 mM SIN-1) or media alone for 72 h. Concentrations of interleukin (IL)-1α, IL-β, IL-1Ra, tumour necrosis factor α (TNFα), IL-6, IL-8, CCL2, IL-10 were measured in culture medium and placental lysates by ELISA and corrected for explant protein content. Data are fold change from control conditions (n = 5–12, line represents median); dashed line represents control values. *p < 0.05, **p < 0.01 Wilcoxon signed rank test.

The same in vitro insults stimulated placental cytokine production and release from placental explants (Fig. 3 and Supplementary Table 3). Oxidative stress induced increased production and the release of IL-1α, IL-1β, IL-6, IL-8 and CCL2 (*p* < 0.05–0.01). There was no effect on the production or release of anti-inflammatory cytokines IL-1Ra or IL-10. A similar pattern but at greater magnitude was detected following low dose LPS treatment, with additional elevation in TNFα and anti-inflammatory IL-1Ra and IL-10 (in lysates and media respectively) (*p* < 0.05–0.01). Hypoxia increased production and release of pro-inflammatory cytokines, with elevated IL-1α, IL-6 and IL-8 (*p* < 0.05–0.01) but only production of IL-1 β and TNFα (*p* < 0.05) was increased. IL-1Ra was unaffected, whilst CCL2 and IL-10 were significantly lower in the medium compared to controls (*p* < 0.01). Nitrative stress had minimal effects on cytokine production or release, except for an inhibitory effect on CCL2 and IL-10 levels in culture medium and IL-6 and IL-8 in lysates (*p* < 0.05–0.01).

### Cellular source of DAMPs in term placenta and explants

To determine the cellular source of DAMPs, immunohistochemistry was performed on term placental villous tissue (uncultured) and placental explants following in vitro treatment (n = 3 placentas/condition).Intracellular uric acid and xanthine oxidase have previously been localised to villous trophoblast, non-villous trophoblast and vessel endothelium in term placenta^38^ but extracellular crystals of monosodium urate act as an inflammatory mediator^39^ therefore staining in explants was not performed in this study.

**Figure 4:**
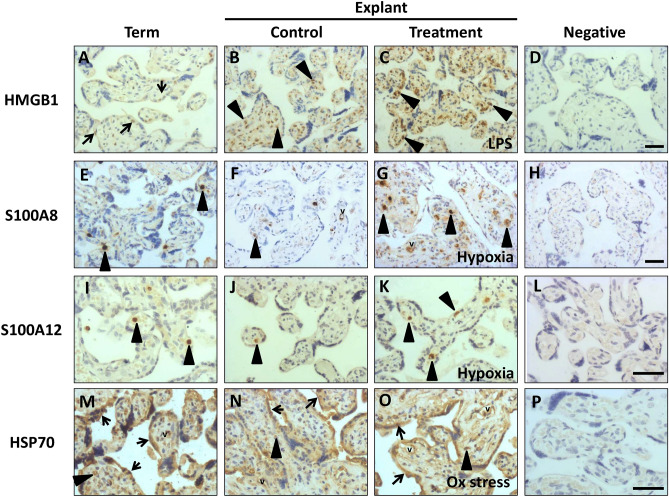
Localisation of DAMPs in term placenta from uncomplicated pregnancies and placental explants cultured under control conditions or exposed to hypoxia (1% O_2_), oxidative stress (1 mM H_2_O_2_) or LPS (1 ng/ml) treatment for 72 h. Representative images from three independent experiments are shown. Immunohistochemistry for: (**A**–**D**) high mobility group box 1(HMGB1), (**E**–**H**) S100 calcium binding protein A8 (S100A8), (**I**–**L**) S100 calcium binding protein A12 (S100A12) and (**M**–**P**) heat shock protein 70 (HSP70). Black arrow heads indicate representative positive stromal cells, black arrows show representative positive trophoblast cells, v = vessels. Magnification × 200. Scale bar marked on negative controls represents 50 μm.

In fresh term placental tissue, faint nuclear HMGB1 immunostaining was present predominantly in trophoblast (Fig. 4A). Culture increased both the number and intensity of positively stained cells, with a shift to staining in the stromal core (Fig. 4B). Under treatment conditions a further elevation in staining intensity and abundance of positive cells was apparent, with staining in both nuclear and cytoplasmic compartments (Fig. 4C, Supplementary Fig. 1B–D).

Both S100 proteins localised predominantly to isolated discrete cells within fetal capillaries and maternal intervillous blood spaces (Fig. 4E,I); these are likely to be fetal and maternal immune cells respectively. Immunostaining patterns of S100A12 were unaltered by explant culture in control conditions or by treatments (Fig. 4J,K), however S100A8 + cells were more frequent under treatment conditions (Fig. 4G, Supplementary Fig. 1F–H) with additional immunostaining observed in capillary endothelial cells following culture (Fig. 4F,G). Cytoplasmic staining for HSP70 was present in trophoblast, individual stromal cells and endothelial cells in uncultured term placenta (Fig. 4M), with no major change following explant culture (Fig. 4N–O, Supplementary Fig. 1M–P). No staining was detected in any negative control samples. Figure 4 provides a subset of images with a full panel in Supplementary Fig. 1 of immunostaining for HMGB1, S100A8, S100A12 and HSP70 in explants cultured under all treatment conditions at higher power magnification.

### Mechanisms underlying DAMP-induced placental inflammation

**Figure 5:**
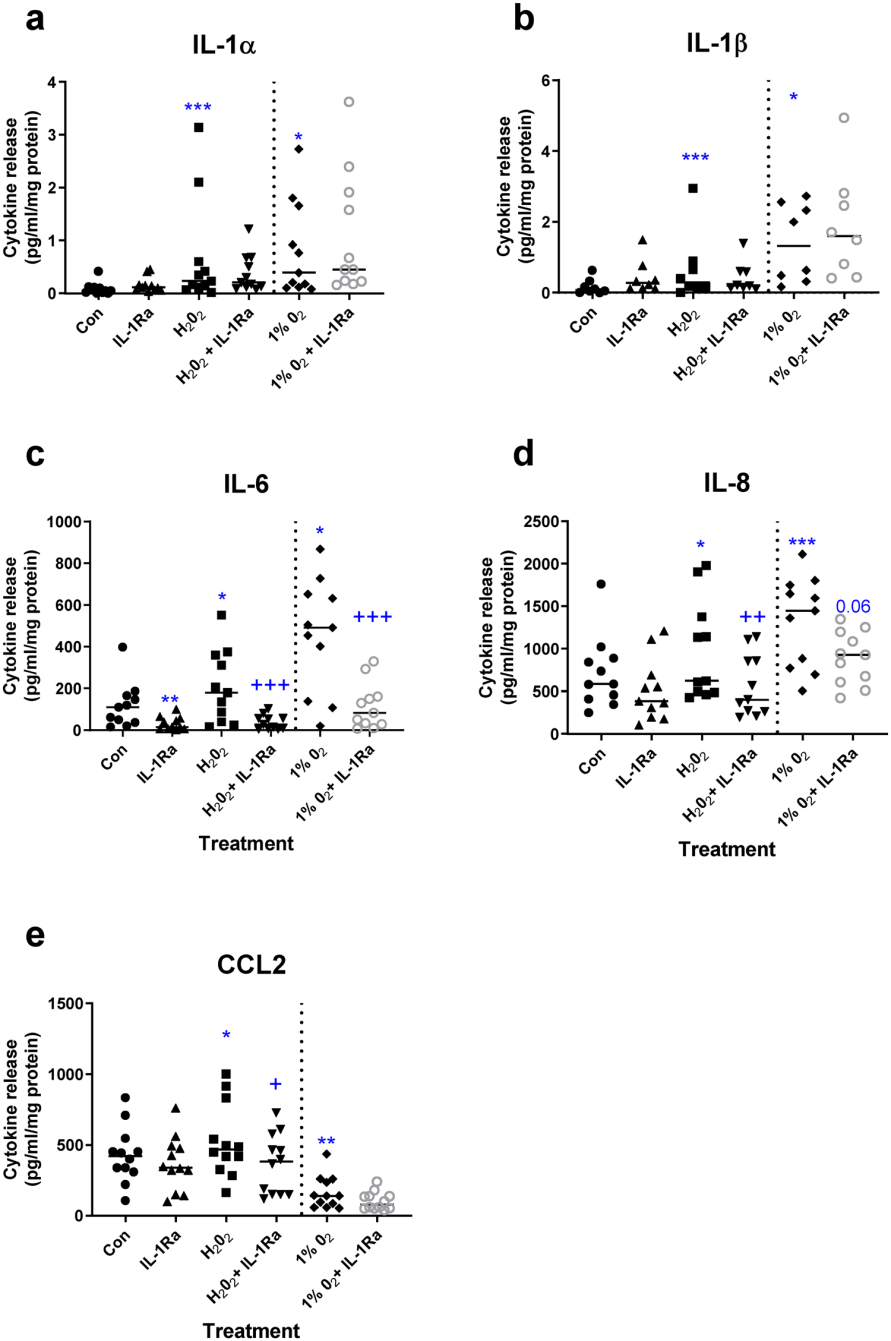
Effect of blockade of interleukin-1 (IL-1Ra) action on cytokine release from placental explants exposed to oxidative stress or hypoxia. Explants were exposed to oxidative stress (1 mM H_2_O_2_), or hypoxia (1% O_2_), in the presence or absence of IL-1 receptor antagonist (IL-1Ra). Cytokines were measured in conditioned medium: (**a**) IL-1α, (**b**) IL-β, (**c**) IL-6, (**d**) IL-8, (**e**) CCL2. Line represents median, n = 8–12. Asterisks represent significant differences compared to control (con) *p < 0.05, **p < 0.01, ***p < 0.001. Plus signs represent significant differences compared to H2O2 or 1%O_2_ alone + *p* < 0.05, +  + *p* < 0.01, +  +  + *p* < 0.001. Friedman with Dunn’s multiple comparison test.

A central role for IL-1 in mediating adverse effects of uric acid has been demonstrated in placenta and other cell types^40^. To determine whether IL-1 action is key to placental inflammation induced by non-pathogenic placental insult, explants were exposed to oxidative stress or hypoxia and treated with IL-1Ra. IL-1Ra treatment had no effect on hypoxia- or oxidative-stress induced IL-1α or IL-1β (Fig. 5a,b). However, IL-1Ra significantly inhibited oxidative stress- and hypoxia-induced IL-6 levels (*p* < 0.001, Friedman test with Dunn’s multiple comparisons test; Fig. 5c). The same effect was observed for IL-8 after treatment with IL-1Ra and oxidative stress (*p* < 0.001), with a similar trend after hypoxia (*p* = 0.06, Fig. 5d). CCL2 levels were inhibited by IL-1Ra under oxidative stress, but not hypoxic conditions (Fig. 5e). IL-1Ra treatment alone had no effect on cytokine release except for an inhibition of IL-6 (*p* < 0.01; Fig. 5c).

**Figure 6:**
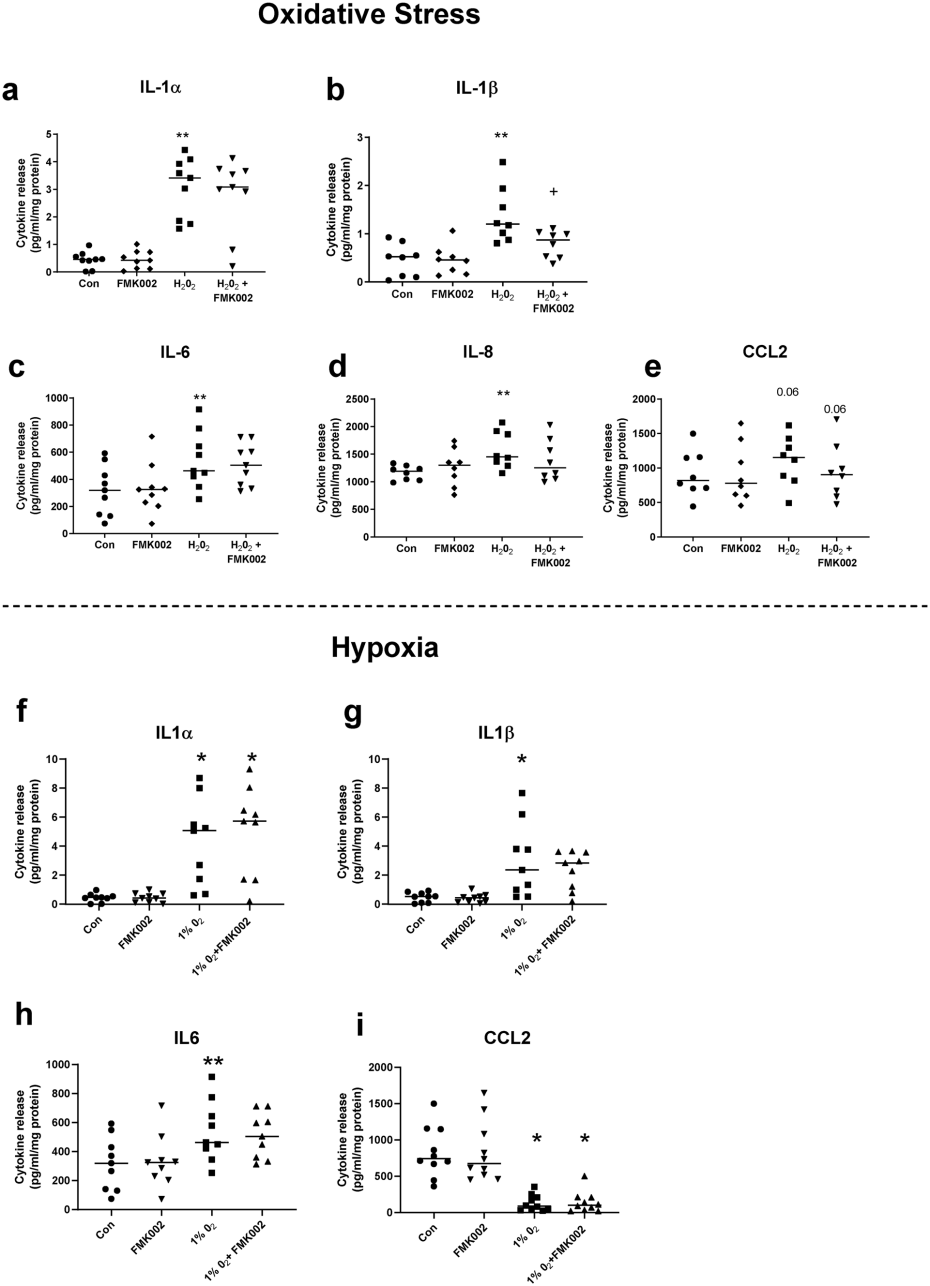
Effect of inhibiting interleukin-1β (IL-1β) activation on cytokine release from placental explants exposed to oxidative stress or hypoxia. Explants were exposed to oxidative stress (1 mM H_2_O_2_), or hypoxia (1%O_2_), in the presence or absence of caspase 1 inhibitor (FMK002) to block inflammasome-processing of pro-IL-1 β. Cytokines were measured in conditioned medium from explants exposed to 1 mM H_2_O_2_: (**a**) IL-1α, (**b**) IL-1β, (**c**) IL-6, (**d**) IL-8, (**e**) CCL2 or 1%O2: (**f**) IL-1α, (**g**) IL-1β, (**h**) IL6, (**i**) CCL2. Line represents median, n = 8–9. Asterisks represent significant differences compared to control (con) *p < 0.05, **p < 0.01. Plus signs represent significant differences compared to H_2_O_2_ or 1%O_2_ alone + *p* < 0.05. Friedman with Dunn’s multiple comparison test.

Similar experiments were performed using a caspase 1 inhibitor (FMK002), to block NLRP3 inflammasome-mediated activation of IL-1β and thereby decipher the role of IL-1β. Oxidative stress-induced IL-1β release was significantly attenuated by FMK002 (*p* < 0.05 Friedman test, Fig. 6b) whilst IL-1α release was unaffected (Fig. 6a). However, this inhibitor had no effect on other cytokines (IL-6, IL-8; Fig. 6c,d), except for a marginal reduction in CCL2 levels following FMK002 treatment (*p* = 0.06; Fig. 6e). Under hypoxia no effect of FMK002 was observed on release of IL1α/β, IL6 or CCL2 (Fig. 6f–i) thus, caspase 1 inhibition had no effect on hypoxia-induced cytokine release.

## Discussion

Here we aimed to conduct a systematic and comprehensive analysis of multiple DAMPs and cytokines produced by the placenta under several stressors present in high-risk pregnancies and to determine whether their release can be reduced by anti-inflammatory treatment. The key findings were increased release of DAMPs uric acid and HMGB1 into culture medium by term placental explants following exposure to oxidative stress and hypoxia. Differential release of the DAMPs cffDNA, S100A8, S100A12 and HSP70 was observed with cffDNA and S100A8 elevated by oxidative stress only and S100A12 and HSP70 release increased under hypoxia. A similar effect was observed in response to low dose LPS as a model of subclinical infection with increased uric acid, HMGB1 and HSP70 release. The same insults induced placental inflammation with increased protein expression and secretion of pro-inflammatory cytokines (IL-1α, IL-1β, IL-6, IL-8), however in the case of CCL2 whilst oxidative stress caused an increase a significant reduction in production and release was seen in explants exposed to hypoxia. Blockade of IL-1 (α and β) action with its antagonist IL-1Ra attenuated the increase in IL-6, IL-8 and CCL2, indicating a coordinating role for IL-1 in inducing downstream placental inflammation following placental exposure to insults. Together these findings support the hypothesis that placental inflammation occurs in response to a placental insult and thus may contribute to placental dysfunction seen in pathological pregnancies.

The significance of the findings that the profile of DAMPs is differentially increased in response to different clinically relevant insults improves understanding of the specific effects these insults induce in the placenta in pathological pregnancies. These insults could all lead to inflammation although different DAMPs and cytokines are induced by each insult. We argue this is clinically relevant as disorders such as FGR are heterogenous in nature, so improved knowledge in underlying mechanisms is crucial in developing individualised treatment strategies for pregnancy complications such as FGR and stillbirth for which currently detection and management remains a major obstetric challenge and to date there are no effective treatments. In vitro studies of the impact of these insults on the human placenta are of high importance to understand the precise mechanisms by which sterile inflammation effects the placenta, which is the first step to determine the potential of anti-inflammatory therapies for pregnancy complications and adverse fetal outcomes. Importantly, the translational significance of this study is that it shows despite the heterogeneous nature of FGR and placental responses to pathologically relevant insults, the potential therapeutic IL1Ra would be effective in blocking proinflammatory cytokine release from different insults examined here.

The DAMPs investigated here were selected based on previous association with placental-mediated pregnancy pathologies, including FGR and stillbirth^41,42^. However, the origin and causes of elevation have not been previously verified. Together with previous data (Supplementary Table 1) our findings support the hypothesis that the placenta is a source of circulating maternal DAMPs and suggests their release occurs in response to placental oxidative stress or hypoxia. A model of subclinical infection (LPS) was included as placental inflammation and elevated DAMPs are frequently detected despite no clinical signs of infection. In these cases, subclinical infection cannot be ruled out but our data support that this may also be a trigger for DAMP release and subsequent exacerbation of placental inflammation.

In some cases, increased DAMP release was accompanied by increased placental intracellular concentrations, implying increased production in response to adverse culture conditions. This was apparent for HMGB1, uric acid and HSP70 in response to oxidative stress. However, uric acid concentrations were lower under nitrative stress, suggesting increased release of intracellular contents, and the same was observed for HMGB1 in response to hypoxia. This may be due to active release, as occurs for HMGB1 in response to hypoxic brain injury^43^ or by activated monocytes^18^. Alternatively, DAMPs may be liberated as a result of cell death and passive release of intracellular contents^17^. However, previous studies using the explant model demonstrate that placental hypoxia and oxidative stress stimulate apoptosis, but not necrosis^34,36^, and necrosis is also less likely as a key mechanism of DAMP release because of the differential patterns detected following different insults, especially for S100A8, S100A12 and HSP70 and lack of LDH release. Moreover, primary trophoblasts exposed to hypoxia in vitro undergo pyroptosis, an inflammatory cell death pathway^44^. Our findings are also consistent with oxidative stress being a major trigger for HMGB1 release^45^ and with reports of increased production and/or release of HSP70 and HMGB1 by placental explants following exposure to oxidative stress^23,26–28^. Uric acid is generated from purines by xanthine oxidase, which is stimulated by stressors including oxidative stress and ischemia^46^. Uric acid has antioxidant properties and hence may have protective actions against free radical damage induced in our placental models, including reducing tyrosine nitrosylation^47^. These actions convert uric acid into a pro-oxidant if not recycled by ascorbate, leading to further oxidative damage and inflammation^48^.

cffDNA was increased in culture medium in response to oxidative stress with a trend to increase under nitrative stress. Maternal plasma circulating cffDNA levels are increased in placental pathologies compared to normal pregnancies including preeclampsia, FGR and reduced fetal movements^10,49^ and a previous study of placental explants exposed to hypoxia for 1 h also saw increased cffDNA release^24^. Although it is currently unclear whether cffDNA may be a cause or consequence of placental dysfunction, mechanistically it can bind to TLR9 receptors present in both trophoblast and Hofbauer cells and elicit production of pro-inflammatory cytokines^50^.

Immunohistochemistry was performed to determine the cellular origin of DAMPs. HMGB1 and HSP70 predominantly originated from trophoblast, with an apparent shift in HMGB1 subcellular localisation and tissue compartment from exclusively nuclear in normal term placenta trophoblast to increasingly cytoplasmic and in the villous stroma following explant culture, suggestive of secretion or release^45^. Both HSP70 and HMGB1 appeared to be upregulated in terms of intensity and number of immunopositive cells following oxidative stress. S100A8 and A12 were almost exclusively localised to immune cells in maternal and fetal circulations; this may account for the considerable variability in measurement of these proteins in lysates. Uric acid was not assessed in this study but previous reports have found expression of its synthetic enzyme xanthine oxidase in trophoblast^20,38^.

Cytokines are produced by resident macrophages, Hofbauer cells, within the placenta, but also by trophoblast^51,52^. In the current study, a prominent feature was the upregulation of pro-inflammatory IL-1α and − β, IL-6 and IL-8 by placental insults. LPS was the only treatment to induce IL-10, consistent with previous observations of differential responses to infectious and non-infectious stimuli^32^. CCL2 is a classic downstream target of IL-1 and was upregulated by oxidative stress and LPS but was markedly inhibited by hypoxia. This response to hypoxia has been previously reported^53^. The patterns of cytokine secretion were strongly mirrored by intracellular cytokine levels, indicating increased expression and confirm a previous finding of increased IL1β production in placental explants exposed for 1 h to hypoxia^25^. Interestingly, nitrative stress induced by SIN1 treatment did not have a pro-inflammatory effect, nor did it lead to DAMP release. However, there were clear effects of the treatment including a reduction in intracellular uric acid, IL6 and IL8 and reduced secretion of CCL2, supporting a shift towards an anti-vs pro-inflammatory balance following nitrative stress in placental explants.

IL-1 plays a pivotal coordinating role in DAMP-induce sterile inflammation (e.g. in gout, stroke, juvenile rheumatoid arthritis and diabetes)^54–56^. We and others have reported a similar mechanistic pathway, i.e. inflammasome-mediated IL-1β secretion, in response to uric acid and HSP70 in placenta^28,40,57^. In the current study, blockade of IL-1 receptor signalling with IL-1Ra treatment inhibited pro-inflammatory cytokine release (IL-6, IL-8 and CCL2) in response to oxidative stress and hypoxia. However, selective blockade of IL-1β maturation with a caspase 1 inhibitor did not induce the same level of cytokine suppression, despite a reduction in mature IL-1β in culture medium being detected indicating effective caspase 1 inhibition. The inhibitors FMK002 and IL1Ra act on totally different parts of the IL1 pathway. FMK002, as a caspase-1 inhibitor, reduced mature IL1β production but had no effect on IL1α release. On the other hand, IL1Ra is an IL-1 receptor antagonist and consequently blocks the actions of both IL-1α and IL-1β. Therefore, in explants treated with FMK002 induction of downstream cytokines was observed, due to IL-1α action. These findings suggest both IL-1α and β are involved in triggering downstream cytokine responses to placental damage and reinforce the use of a therapeutic strategy targeting the IL-1 receptor with further mechanistic analyses required to elucidate roles of individual IL-1 ligands.

A limitation of the placental explant model is high degree of variability, due in part to differences in explant composition (particular in relation to immune cell numbers), and other known confounders (e.g. maternal adiposity, extent of preparation for labour, maternal age, smoking) that may affect inflammatory status^58–60^. These were limited as far as possible by strict inclusion criteria, but eliminating interpatient variability is impossible in human studies, hence statistical analyses were used to normalize to each control sample. Further studies of isolated cytotrophoblast cells would enable analysis of effects on trophoblast alone, but for the current study it was important to use a more physiological model to study the effects of these insults with all cell types and interactions present^61^. Both the hypoxia and oxidative stress models employed are relatively crude and extreme models, but have been well characterised and mimic the placental dysfunction detected in pregnancy pathologies^33,34,36^. These provide proof of concept that placental insults can cause DAMP release within a relatively short treatment window.

The current study provides evidence that DAMPs and inflammatory cytokines are induced in response to placental insult and provides a potential explanation for their elevation in pregnancy complications such as FGR, stillbirth and pre-eclampsia^10,11,42,49,62–68^. The impact of DAMPs and these cytokines on the placenta is not fully understood. We previously demonstrated adverse effects of uric acid and downstream mediator IL-1 on trophoblast differentiation and survival in vitro and a negative effect on fetal growth in a rat model^40^. Previous studies have shown uric acid and pro-inflammatory cytokines (IL-6, TNF, IL-1) can modulate placental nutrient transport and induce apoptosis^69,70^.

We argue that these findings support the hypothesis that primary placental insults (hypoxia and oxidative stress) lead to a shift in the pro vs anti-inflammatory balance within placental explants, which then prompts placental inflammation seen in cases of FGR and stillbirth, which may be clinically evident in villitis of unknown etiology (VUE)^11^. More detailed analyses of the effects of DAMPs and cytokines on placental function are required to ascertain a causal role for inflammation as a “second hit” leading to pregnancy pathologies and adverse neonatal outcomes. Future work on production and transport of DAMPs to dissect the origins of increased intracellular concentrations is required as is identifying other cell death pathways such as pyroptosis and necroptosis. Whether IL1Ra is effective in reducing placental damage and improving explant viability and further dissection of feedback loops is desirable. Deciphering the mechanisms, particularly verifying a coordinating role for IL-1, is important in terms of clinical application as therapeutic targeting of IL-1 shows promise in protecting against adverse fetal outcomes in rodent models of infection-induced placental inflammation^71,72^. Similar investigations are warranted into the potential benefit of targeting sterile placental inflammation.

## Materials and methods

Unless otherwise stated materials were supplied by Sigma-Aldrich, Dorset, UK.

### Placental collection and explant culture

Normal term placentas (37–42 weeks gestation) from women undergoing Cesarean section for a singleton pregnancy were collected and sampled within 30 min after delivery. Exclusion criteria were maternal BMI < 18.5 or > 30 kg/m^2^, maternal age > 40 years, evidence of maternal or intra-uterine infection, diabetes, hypertension or fetal anomalies. Full thickness villous biopsies 1 cm^3^ were excised using a systematic uniform random sampling system from 4 locations on the placenta as recommended by Burton et al.^73^. Three explants approximately 5 mg were excised, rinsed and randomly placed in netwells (15 mm diameter, 74 µm mesh, Corning) supported in a 12-well plate containing 1.5mls of CMRL‐1066 culture medium (Gibco, Paisley, UK) supplemented with 5% heat‐inactivated fetal bovine serum (Gibco, Paisley, UK), 100 IU/ml penicillin, 100 μg/ml streptomycin, 1 μg/ml insulin, 0.1 μg/ml hydrocortisone, and 0.1 μg/ml retinol acetate^61^. Explants were incubated at 37 °C in a humidified chamber in a 5% CO_2_/95% air gas mixture.

### Explant culture: insult models and treatments

Term placental villous tissue maintained in explant culture is a well-established model^61^which has been used to study placental hypoxia and oxidative/nitrative stress^32–35^. Doses of reagents used were decided based on previously well characterised data showing that they were physiologically relevant, tissue integrity was maintained, and phenotypic and functional changes were induced previously observed in vivo in FGR placentas^34,74^. Treatments were applied 24 h after plating out of explants (designated day 1) at a final concentration of 1 mM H_2_O_2_, 1 mM 3-morpholino-sydnonimine (SIN-1), or exposure to 1% O_2_ (1% O_2_/94% N_2_/5% CO_2_) in a hypoxic incubator and changed every 24 h for 3 days. A further set of explants were treated with low dose LPS (1 ng/ml) to mimic subclinical infection. For mechanistic studies, treatment with recombinant human IL1Ra at 1 μg/ml or the caspase 1 inhibitor Z-WEHD-FMK (FMK002) at 10 μM (R and D Systems, Abingdon, UK) was initiated at the same time as the insults with sterile PBS or DMSO (0.05%) as vehicle controls respectively. The concentration of inhibitors IL1Ra and FMK002 chosen have previously been shown to be effective at attenuating inflammation and cell death in placental explants in culture^32,40^. Media and tissue samples were collected and stored for analysis on day 4. This time point was chosen as this allowed cytokine levels to recover to baseline following explant dissection and culture and a functional syncytium has been formed as indicated by hCG secretion^61^. Media were stored at -20 °C and explants were snap frozen or fixed in 10% formalin.

### Assessment of tissue viability

To ensure explant tissue viability was maintained under experimental conditions markers of apoptotic cell death (M30), cell proliferation (Ki67), release of the placental hormone β-human chorionic gonadotropin (βhCG, functional marker) and necrosis (LDH release) were measured. Immunohistochemistry for M30 and Ki67 on FFPE explants was performed as described previously^32^ Mouse monoclonal primary antibodies used to detect proliferation and apoptosis were anti-Ki67 (0.16 µg/mL, Dako, Ely, UK) and anti-M30 (66 pg/mL, Roche, Hertfordshire, UK), respectively, with nonimmune mouse IgG as a negative control. Image capture of 10 random fields of view (FOV) per section was performed on an Olympus BX41 microscope with a QICAM Fast 1394 camera (QImaging, Canada) and Image Pro Plus 7.0 software (Media Cybernetics, UK). Proliferative and apoptotic indices were calculated by dividing Ki67 + or M30 + cells by total nuclei in each FOV using Histoquest Tissue Analysis software v3.0 (https://tissuegnostics.com/products/single-cell-analysis/histoquest) (TissueGnostics GmBH, Vienna, Austria). Conditioned explant media were analysed for β-hCG by ELISA (DRG Diagnostics, IDS, Tyne and Wear, UK) and for LDH activity (Roche cytotoxicity detection kit) to assess necrosis following the manufacturer’s instructions. Media analyses were corrected for explant protein content.

### Protein extraction and analysis

Explants were homogenised using a Stuart SH1 polytron probe at 35,000 rpm for 30 s on ice in 0.5 ml of lysis buffer (phosphate-buffered saline containing 1% Triton X-100 plus protease inhibitor cocktail (Calbiochem, Watford, UK). Homogenates were centrifuged at 12,500 g for 10 min at 4 °C and lysates frozen at − 80 °C until required. Protein concentration was determined using the Pierce BCA protein assay kit (ThermoFisher Scientific, UK).

### Measurement of DAMPs

Uric acid concentrations in media and explant lysates were measured using the Quantichrom Uric Acid assay kit (Universal Biologicals, Cambridge, UK). HMGB1 concentrations were determined using a specific ELISA (IBL International, Oxford Biosystems Ltd, Abingdon, UK). The proteins S100A8, S100A12 and HSP70 were all detected using Duoset ELISA kits (R and D Systems, Abingdon, UK) following the manufacturer’s protocol. Media and lysate concentrations were corrected for protein concentration determined by BCA assay (mg/ml). The intra assay variability was 3.6–7.9% and the inter assay variability was 5.5–9.8%.

### Cytokine ELISAs

Cytokines (IL1α, IL1β, IL1Ra, TNFα, IL6, IL8, CCL2 and IL10) in media and explant lysates were measured using Duoset ELISA kits (R and D Systems, Abingdon, UK) following the manufacturer’s protocol. Media concentrations were corrected for explant wet weight (g) and lysates for protein concentration determined by BCA assay (mg/ml). The intra assay variability was 4.3–8.2% and the inter assay variability 6.3–10.2%.

### Nitrotyrosine ELISA

To confirm 1 mM SIN1 was inducing explant nitrative stress, detection of 3-nitrotyrosine-containing proteins generated by SIN1 peroxynitrite production in culture was quantified using a specific ELISA (OxiSelect Nitrotyrosine ELISA, Cell Biolabs, Cambridge Bioscience, Cambridge, UK). Protein lysates from matched control and SIN1 exposed explants were analysed according to manufacturer’s instructions and corrected for lysate protein concentration.

### Isolation and quantification of cffDNA from explant media

Isolation of cffDNA released by explants was carried out using the QIAamp Blood Mini Kit (Qiagen Ltd, Manchester, UK) starting with equal volumes of frozen culture media. The manufacturer’s protocol for purification from blood or body fluids was followed. Concentration of the DNA (ng/ul) was determined on a Nanodrop 2000 Spectrophotometer (Thermo Scientific, Loughborough, UK). Samples were corrected for total explant protein per well and expressed as fold change from experimental control.

### Immunohistochemistry for DAMPs

Sections 5 μm thick were prepared from formalin fixed paraffin embedded blocks of normal term placenta from uncomplicated pregnancies (accessed from the MFHRC Biobank (08/H1010/ + 55)and explants exposed to treatments. Heat mediated antigen retrieval using 0.01 M sodium citrate buffer pH 6 was performed and endogenous peroxide quenched with 3% H_2_O_2_. Primary antibodies (HMGB1 at 4 μg/ml, Novus Biologicals, UK (NB100-2322); S100A8, 5.8 μg/ml, Abcam, UK (ab180735); S100A12, 2 μg/ml, Abcam UK (ab196740); HSP70, 10 μg/ml, Abcam, UK (ab2787) were applied at 4 °C for 18 h with equivalent dilutions of nonimmune rabbit or mouse IgG as negative controls. Secondary antibodies were applied for 30 min at room temperature, either biotinylated goat anti-rabbit or goat anti-mouse (DAKO, Agilent Technologies, Cheadle, UK) and the signal amplified with avidin-peroxidase (5 μg/ml). For colour development the chromogenic substrate diaminobenzidine (DAB) was used followed by counterstaining in Harris’ hematoxylin and mounting in DPX.

### Image analysis

Immunostained sections were imaged to determine the cellular and subcellular localisation of DAMPs in term uncultured placenta and to assess any differences following culture in the presence and absence of treatments. Image analysis was performed on an Olympus BX41 microscope with a QICAM Fast 1394 camera (QImaging, Canada) and Image Pro Plus 7.0 software (Media Cybernetics, UK). All images were taken randomly across the sections at × 20 and × 40 magnification.

### Data analysis

Statistical analyses were performed using GraphPad Prism (version 8.04). To account for inter-placental variability, fold change comparisons were made between control and treated explants for DAMP and cytokine measurements and Wilcoxon signed-rank analyses performed^75^. Placental treatments with anti-inflammatory mediators were analysed using Friedman with Dunn’s post-hoc test. Results were considered statistically significant if *p* < 0.05.

### Ethical approval and informed consent

This study was performed with local research ethics committee approval (North West Regional Ethics Committee 08/H1010/55+5) and in accordance with relevant guidelines and regulations. Informed written consent from all participants was obtained during pregnancy prior to collection of samples.

## Supplementary Information

Supplementary Information
