# Supplementary material for: Hypoxia and oxidative stress induce sterile placental inflammation in vitro
Source: Sci Rep. 2021 Mar 31;11:7281. doi: 10.1038/s41598-021-86268-1 (PMC8012380; doi:10.1038/s41598-021-86268-1)
Supplement: Supplementary file 1 — Supplementary Information [file 41598_2021_86268_MOESM1_ESM.pdf]

Supplementary Information File

Hypoxia and Oxidative Stress Induce Sterile Placental Inflammation In Vitro

Bernadette C. Baker*[1], Alexander E. P. Heazell[1,2], Colin Sibley[1,2], Rachael Wright[1], Helen Bischof[1,2], Frances Beards[1,2], Tatiana Guevara[1,2], Sylvie Girard[3], Rebecca L. Jones[1,2]

[1]Maternal and Fetal Health Research Centre, Division of Developmental Biology and Medicine, School of Medical Sciences, Faculty of Biology, Medicine and Health, University of Manchester, Manchester Academic Health Science Centre; [2]St Mary's Hospital, Central Manchester NHS Foundation Trust, Manchester Academic Health Science Centre; Manchester M13 9WL, UK. [3]Fetomaternal and Neonatal Pathologies Research Axis, Sainte-Justine Hospital Research Center; Department of Obstetrics and Gynecology, Universite de Montreal, Montreal, Quebec, Canada.

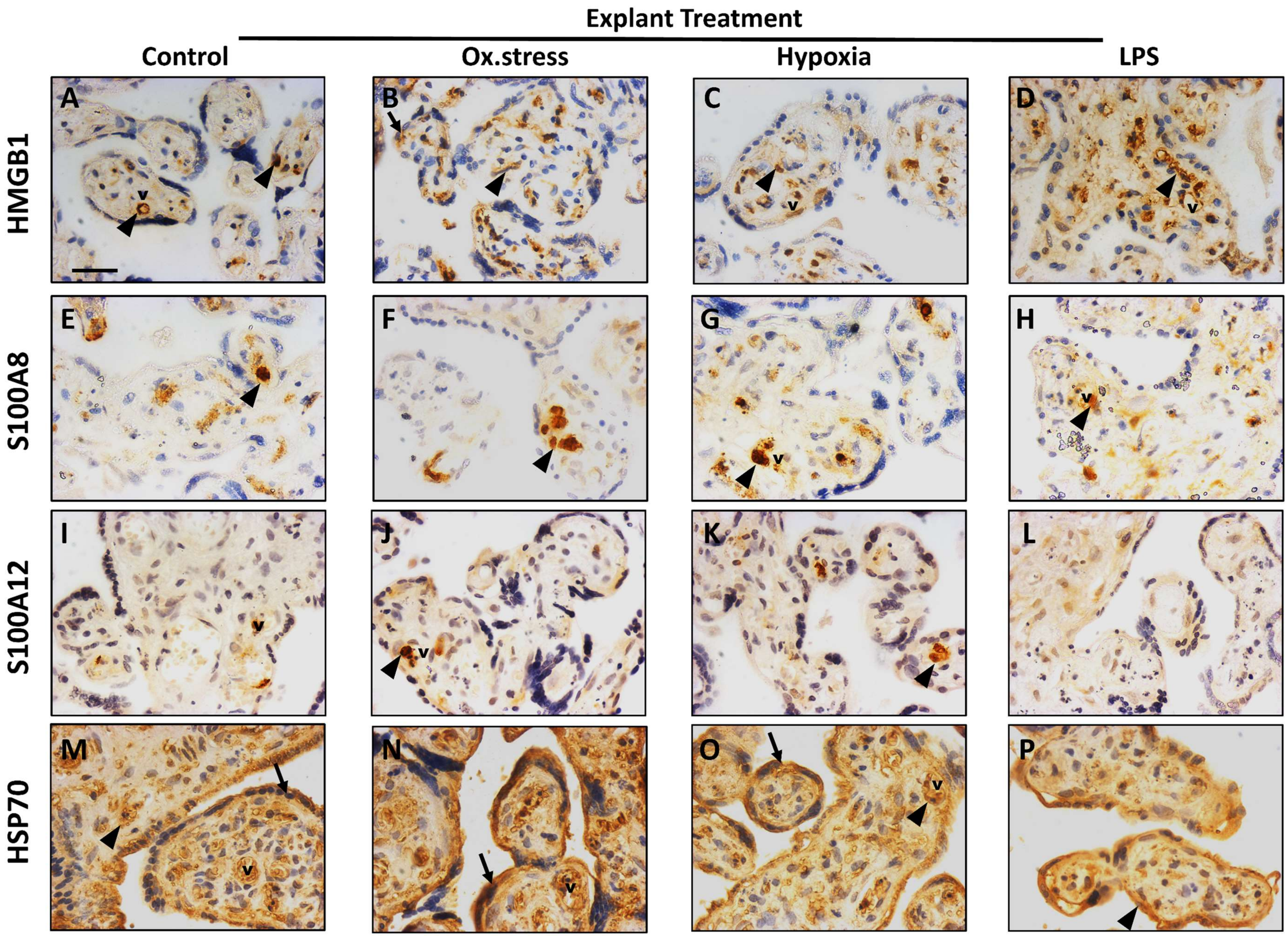

Supplementary Figure 1. **Localisation of DAMPs in placental explants cultured under control conditions or exposed to hypoxia (1% O_2), oxidative stress (1mM H_2O_2) or LPS (1ng/ml) treatment for 72h.** Representative images from three independent experiments are shown. Magnification x400. Scale bar marked on panel A represents 25µm. Black arrow heads indicate representative positive stromal cells, black arrows show representative positive trophoblast cells, v = vessels.

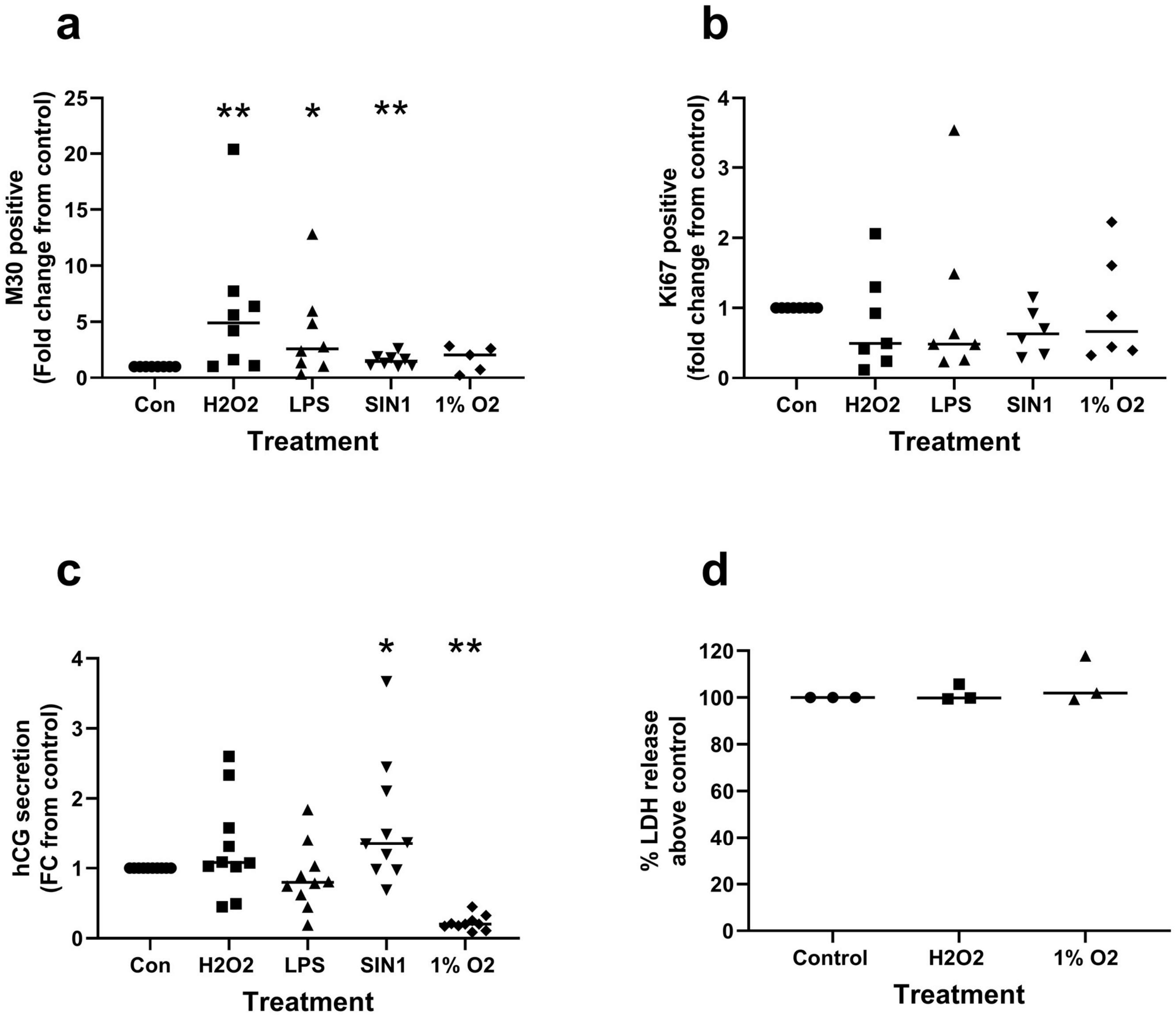

Supplementary Figure 2. Markers of tissue viability measured in placental explants exposed for 72hr to oxidative stress (H_2O_2), LPS, nitrative stress (SIN1) and hypoxia (1% O_2). Placental explants were examined for markers of cell apoptosis and proliferation by IHC, hormone secretion by ELISA and cell necrosis using an LDH assay a) M30+ cells, n=5-8 placentas b) Ki67+ cells, n=6-7 placentas c) hCG secretion, n=10 placentas d) LDH release, n=3 placentas. All data were normalised to control. Line represents media, *p<0.05, **p<0.01 Wilcoxon signed rank test.

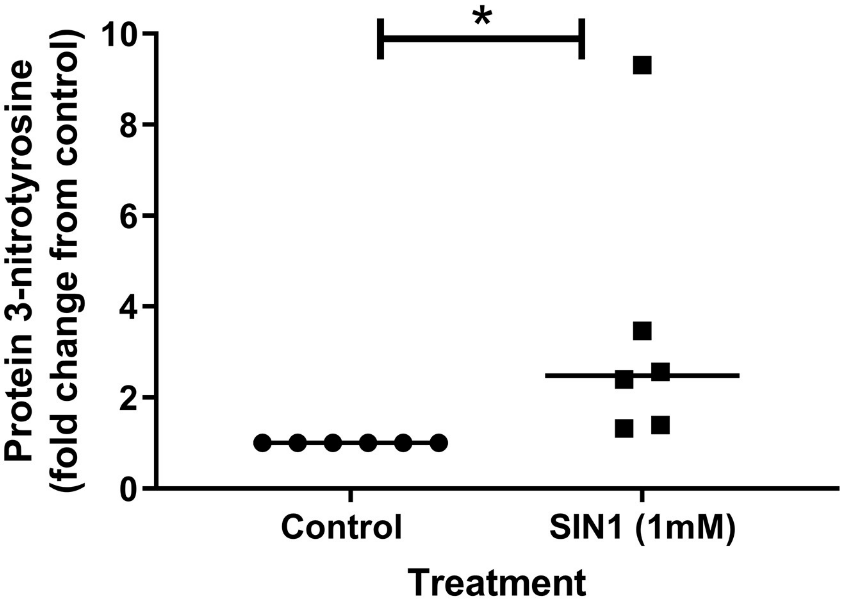

Supplementary Figure 3. Production of protein 3-nitrotyrosine residues in placental explants exposed to 1mM SIN1. Cell lysates from explants were quantitated by ELISA for 3-nitrotyrosine in proteins. n=6 placentas. Data were expressed as fold change from control. Line represents median, *p<0.05 Wilcoxon signed rank test.

Study authors	Model	Conditions	DAMP/Cytokine investigated	Findings
Tjoa et al, 2006 [1]	Term villous explants (20h culture)	Hypoxia/reoxygenation (H/R) (0.5% for 1hr followed by normoxia (10%) for 6 or 19h +/- vit C or E	cfDNA	cell-free DNA in supernatant significantly ↑ at 20h hours after H/R.<br>Associated with ↑ STB apoptosis and necrosis.<br>cell-free DNA release and apoptosis significantly ↓ by antioxidant vitamins C and E
Cindrova-Davis et al, 2007 [2]	Term villous explants (16h culture)	Hypoxia/reoxygenation (0.5% for 1hr followed by normoxia (10%) for 6 or 15h +/- vit C or E or p38 inhibitor	TNFα/IL1β	p38 MAPK and NFkB pathways activated. Oxid stress markers detected<br>↑ release and tissue content TNFα and tissue content only of IL1β, release not measured<br>↑ COX-2 protein expression<br>↑ apoptosis. Vit C and E blocked all these effects
Tskitishvili et al, 2010 [3]	Term villous explants and amnion 2 or 6h	Oxidative stress (HNE, NaF, X/XO)	S100B	↑ S100B release from villous explants and amnion cultures
Jiang et al, 2014 [4]	1st trimester primary mouse trophoblast JEG-3 cells	Hypoxia (1% O_2) vs 21% Up to 48h	HMGB1	↑mRNA at 24h in PMT+JEG3 in hypoxia<br>↑release and intracellular protein at 24 and 48h hypoxia both cell types
Collett et al, 2018 [5]	BeWo cells	ER stress (Tunicamycin or Thapsigargin)	HMGB1/HSP70/Histone H3 (EV release only)	↑ EV-associated high mobility group protein B1 (HMGB1), heat shock protein 70 (hsp70) and histone H3 released from severely ER-stressed cells<br>Antioxidant PTDC abolished EV associated DAMP release
Nunes et al, 2018 [6]	Term villous explants (24h stabilisation followed by 4 or 24h treatment)	H_2O_2 (100 or 1000μM) +/- Glybenclamide (NLRP3 inflammasome blocker)	HSP70/IL1β/TNFα/IL10	↑ Release HSP70/IL1β/hCG/SOD/Catalase at 1mM<br>↑ mRNA caspase 1/IL1β/TNFα<br>↓ mRNA and protein IL10 release<br>Glybenclamide reduced NLRP3 + IL1β mRNA and caspase 1 release

Supplementary Table 1. Summary of previous placental *in vitro* studies on DAMPs/Cytokines

Treatment	Control (H$_2$O$_2$, LPS, SIN-1)	Oxidative stress (1mM H$_2$O$_2$)	LPS control (1ng/ml)	Nitrative stress (1mM SIN-1)	Control (Hypoxia only)	Hypoxia (1% O$_2$)
Uric acid (M) [µM/mg]	20.6 (11 – 42.1)	18.4 (16 – 48.6) *	20.3 (16.4 – 71.6) *	25.1 (15.9 – 35.8)	0.22 (0.16 – 2.9)	0.94 (0.2 – 5.5) *
Uric acid (L) [µM/mg]	16.7 (4.4 – 80.9)	50.3 (16 – 48.6)	38.7 (30.1 – 68)	3.2 (1.3 – 5.2) *	29.4 (12.4 – 60.2)	29.7 (16.6 – 50.6)
HMGB1 (M) [ng/ml/mg]	6.6 (2.3 – 143)	20.1 (7.6 – 179) **	32.8 (7.3 – 273) **	4.3 (0.87 – 12.2)	5.4 (3.2 – 26.8)	14 (8 – 27) *
HMGB1 (L) [ng/ml/mg]	147 (99.5 – 318)	265 (96 – 414) *	181 (89 – 350)	125 (102 – 162) **	507 (269 – 949)	241 (79.9 – 809) **
cffDNA (M) [ng/µl/mg]	4.5 (1.6 – 10.4)	4.7 (2.9 – 14.8) *	3.3 (2.3 – 18.4)	10.5 (835 – 14.0)	3.1 (1.6 – 5.3)	2.6 (1.9 – 4.0)
S100A8 (M) [pg/ml/mg]	1.3 (0.16 – 10.4)	4.5 (0.6 – 40.4) **	4.3 (1.4 – 23) *	0.7 (0.3 – 1.7)	0.17 (0.06 – 0.25)	0.2 (0.04 – 0.3)
S100A8 (L) [pg/ml/mg]	288 (126 – 761)	290 (132.5 – 669)	253 (181 – 320)	359 (220 – 695)	1069 (238 – 1461)	960 (200 – 2182)
S100A12 (M) [pg/ml/mg]	105 (6.7 – 393)	113.5 (63.6 – 320.1)	156 (52.3 – 451)	67.9 (49.5 – 154)	6.8 (4 – 7.25)	7.86 (4.4 – 13) *
S100A12 (L) [pg/ml/mg]	2.0 (0.34 – 47.9)	1.02 (0.19 – 3.1)	0.55 (0.23 – 4.3)	4.3 (0.7 – 13.9)	20.4 (0.85 – 47.7)	24 (0.4 – 57.5) *
HSP70 (M) [ng/ml/mg]	4.9 (0.3 – 35.9)	7.6 (3.0 – 58.2)	10 (2.4 – 41.2) *	6.31 (4.8 – 8.2)	0.29 (0.16 – 0.37)	0.45 (0.22 – 0.58) **
HSP70 (L) [ng/ml/mg]	16.3 (7.7 – 23.6)	14.3 (8.5 – 27)	16.2 (13.2 – 27.8)	16.1 (13.5 – 18.9)	28.1 (16.3 – 39.9)	27.8 (8.2 – 70.1)

Supplementary Table 2: Summary of absolute DAMPs concentrations detected in media and lysates of placental explants treated with pathological insults. (M) denotes media and (L) tissue lysate. Values are Median with range bracketed. Stars denote level of significance (*$p<0.05$, **$p<0.01$; Wilcoxon signed rank test) as determined once normalised to control.

Treatment	Control (H_2O_2, LPS, SIN-1)	Oxidative stress (1mM H_2O_2)	LPS (1ng/ml)	Nitrative stress (1mM SIN-1)	Control (Hypoxia only)	Hypoxia (1% O_2)
IL1α (M)	1.5 (0.16 – 48.8)	7.9 (1.2 – 55.3) **	17.6 (2.1 – 143.5) **	0.81 (0.1 – 1.75)	1.6 (0.37 – 8.7)	8 (0.76 – 27.8) **
IL1α (L)	46.5 (0.9 – 136.4)	7.6 (2.0 – 249.5) **	10.8 (6.7- 369.2) **	80.2 (25 – 155.9)	166.3 (79.5 – 537)	387.4 (114.6 – 1160) *
IL1β (M)	0.38 (0.09 – 21.4)	0.94 (0.18 – 53.8) *	4.2 (1.3 – 90) **	0.2 (0.03 – 0.64)	5.0 (0.9 – 12.2)	6.6 (0.7 – 38.1)
IL1β (L)	10.1 (1.5 – 34.1)	12.3 (3.6 – 70.2) *	23.1 (12.9 – 91.2) **	17.4 (5.8 – 26.5)	148 (50.5 – 383)	332.8 (73.9 – 758) *
IL1Ra (M)	114 (5.4 – 305.1)	144.6 (28.6 – 507.8)	239 (54 – 910)	8.7 (1.75 – 65.3)	121 (61.8 – 539)	180.6 (56.3 – 542.6)
IL1Ra (L)	2,582 (659 – 6,188)	3,154 (645 – 32,957)	8,021 (630 – 49,638) **	1,261 (338 – 1,788)	16,291 (12,521 – 44,142)	14,604 (7,613 – 52,678)
TNFα (M)	3.6 (0.3 – 323)	8.5 (2.2 – 134)	86.3 (8.8 – 550) **	2.0 (0.75 – 3.6)	5.5 (1.7 – 24.3)	4.1 (1.2 – 45.2)
TNFα (L)	112 (8.3 – 281)	78.8 (6.6 – 499) *	259 (17.3 – 738) **	160.5 (4.5 – 312)	10.5 (4.8 – 14.1)	15.9 (6.1 – 23.6) *
IL6 (M)	1,277 (439 – 1,061)	2,702 (1,412 – 14,850) **	5,627 (1,485 – 10,011) **	1,009 (723 – 1,767)	2,210 (958 – 4,128)	4,094 (1,570 – 5,630) *
IL6 (L)	290 (51.1 – 1,061)	236 (103 – 911) **	191 (141 – 1,444) *	425 (142 – 612) **	1,047 (256 – 1,660)	2,043 (843 – 3,210) *
IL8 (M)	5,263 (3,289 – 11,815)	7,260 (3,689 – 10,810) *	5,750 (3,418 – 7,586) *	4,669 (3,399 – 5,749)	1,204 (509.5 – 1651)	1,562 (781 – 2,772) *
IL8 (L)	1,616 (1,259 – 3,130)	2,563 (1,217 – 3,814) *	2,127 (1,198 – 2,733)	1,411 (826 – 1,651) *	7,662 (3,000 – 12,463)	9,792 (3,761 – 25,615) **
CCL2 (M)	7,605 (546 – 15,403)	7,731 (550 – 22,239) *	9,221 (5,042 – 24,730) *	5,505 (2,621 – 9,056) **	760 (302 – 1,191)	209 (94 – 651) **
CCL2 (L)	3,620 (1,532 – 7,329)	6,227 (1,919 – 14,646) **	7,108 (2,437 – 12,785) **	2,435 (1,224 – 2,849)	4,021 (2,668 – 6,455)	2,287 (1,331 – 4,179)
IL10 (M)	38.8 (9.9 – 75.6)	41.3 (16 – 127)	89.9 (37.5 – 282) **	29.3 (12.3 – 37) **	0.98 (0.7 – 1.69)	0.55 (0.22 – 1.12) **
IL10 (L)	456 (158 – 1,919)	395 (109 – 999)	370 (113 – 776)	1,031 (157 – 1,530)	1,220 (65 – 2,471)	1,482 (43 – 3,142)

Supplementary Table 3: Summary of absolute cytokine concentrations detected in media and lysates of placental explants treated with pathological insults. (M) denotes media and (L) tissue lysate. Values are Median with range bracketed All cytokine concentrations are pg/ml/mg protein. Stars denote level of significance (*p<0.05, **p<0.01; Wilcoxon signed rank test) as determined once normalised to control.

References

1 Tjoa, M. L., Cindrova-Davies, T., Spasic-Boskovic, O., Bianchi, D. W. & Burton, G. J. Trophoblastic oxidative stress and the release of cell-free feto-placental DNA. *The American journal of pathology* **169**, 400-404, doi:10.2353/ajpath.2006.060161 (2006).

2 Cindrova-Davies, T., Spasic-Boskovic, O., Jauniaux, E., Charnock-Jones, D. S. & Burton, G. J. Nuclear factor-kappa B, p38, and stress-activated protein kinase mitogen-activated protein kinase signaling pathways regulate proinflammatory cytokines and apoptosis in human placental explants in response to oxidative stress: effects of antioxidant vitamins. *The American journal of pathology* **170**, 1511-1520, doi:10.2353/ajpath.2007.061035 (2007).

3 Tskitishvili, E. *et al.* Oxidative stress-induced S100B protein from placenta and amnion affects soluble Endoglin release from endothelial cells. *Molecular human reproduction* **16**, 188-199, doi:10.1093/molehr/gap104 (2010).

4 Jiang, R. *et al.* Hypoxic trophoblast HMGB1 induces endothelial cell hyperpermeability via the TRL-4/caveolin-1 pathway. *Journal of immunology* **193**, 5000-5012, doi:10.4049/jimmunol.1303445 (2014).

5 Collett, G. P., Redman, C. W., Sargent, I. L. & Vatish, M. Endoplasmic reticulum stress stimulates the release of extracellular vesicles carrying danger-associated molecular pattern (DAMP) molecules. *Oncotarget* **9**, 6707-6717, doi:10.18632/oncotarget.24158 (2018).

6 Nunes, P. R. *et al.* Hydrogen peroxide-mediated oxidative stress induces inflammasome activation in term human placental explants. *Pregnancy hypertension* **14**, 29-36, doi:10.1016/j.preghy.2018.07.006 (2018).
